# The Effects of Swelling and Porosity Change on Capillarity: DEM Coupled with a Pore-Unit Assembly Method

**DOI:** 10.1007/s11242-016-0689-8

**Published:** 2016-04-23

**Authors:** Thomas Sweijen, Ehsan Nikooee, S. Majid Hassanizadeh, Bruno Chareyre

**Affiliations:** Department of Earth Sciences, Environmental Hydrogeology Group, Utrecht University, Princetonplein 9, 3584CC Utrecht, The Netherlands; 3SR, University Grenoble-Alpes, 38000 Grenoble, France; 3SR, CNRS, 38000 Grenoble, France; Department of Civil and Environmental Engineering, School of Civil and Material Engineering, Shiraz University, Shiraz, Iran

**Keywords:** Discrete Element Method, Swelling porous media, Capillary pressure–saturation, Hydromechanical coupling, Absorbent gelling material (AGM), Porosity change

## Abstract

In this study, a grain-scale modelling technique has been developed to generate the capillary pressure–saturation curves for swelling granular materials. This model employs only basic granular properties such as particles size distribution, porosity, and the amount of absorbed water for swelling materials. Using this model, both drainage and imbibition curves are directly obtained by pore-scale simulations of fluid invasion. This allows us to produce capillary pressure–saturation curves for a large number of different packings of granular materials with varying porosity and/or amount of absorbed water. The algorithm is based on combining the Discrete Element Method for generating different particle packings with a pore-unit assembly approach. The pore space is extracted using a regular triangulation, with the centres of four neighbouring particles forming a tetrahedron. The pore space within each tetrahedron is referred to as a pore unit. Thus, the pore space of a particle packing is represented by an assembly of pore units for which we construct drainage and imbibition capillary pressure–saturation curves. A case study on Hostun sand is conducted to test the model against experimental data from literature and to investigate the required minimum number of particles to have a Representative Elementary Volume. Then, the capillary pressure–saturation curves are constructed for Absorbent Gelling Material particles, for different combinations of porosity values and amounts of absorbed water. Each combination yields a different configuration of pore units, and thus distinctly different capillary pressure–saturation curves. All these curves are shown to collapse into one curve for drainage and one curve for imbibition when we normalize capillary pressure and saturation values. We have developed a formula for the Van Genuchten parameter $\alpha$ (which is related to the inverse of the entry pressure) as a function of porosity and the amount of absorbed water.

## Introduction

The swelling and the deformation of granular porous media under variably saturated conditions are a common phenomenon in geotechnical problems, product engineering, and biological tissues. Examples are swelling clays (Murad and Cushman 1996; Bennethum and Cushman 1996; Romero et al. 2011), foods (Takhar 2014), paper and tissues (Sun et al. 2014; Qin and Hassanizadeh 2014), biopolymers (Singh et al. 2003; Malakpoor et al. 2007), and absorbent polymers in hygienic products (Diersch et al. 2010). Numerical models have been employed to study the swelling and the subsequent deformation of granular materials. An example is a model for the swelling of hydrogels that is based on the exploitation of the entropy inequality by Huyghe and Janssen (1997), which was validated against experiments by Frijns et al. (1997). Also, a numerical model was developed by Diersch et al. (2010) for the simulation of partially saturated swelling granular materials, such as Absorbent Gelling Material (AGM) particles used in hygienic products. That model employs empirical equations to account for the effect of swelling and the subsequent deformation on hydraulic parameters such as capillary pressure–saturation curve, permeability, and porosity.

It is well known that the presence of pore water inside a porous medium affects the behaviour of the porous medium during deformation and that the deformation affects hydraulic properties of a porous medium. For instance, the presence of liquid bridges between grains increases the cohesion in a granular material and therefore stiffens the material (Lu et al. 2007; Scholtès et al. 2009). Moreover, any change in the structure of the porous medium causes a change in its permeability, e.g. lowering the porosity lowers the permeability (Bear 1972; Torskaya et al. 2014). The interaction between pore fluids and the solid grains and its subsequent effect on the mechanical behaviour of the medium is referred to as hydromechanical coupling (Tarantino and Tombolato 2005; Simms and Yanful 2005; Nikooee et al. 2013; Choo et al. 2015). A key ingredient of the hydromechanical coupling of partially saturated porous media is the capillary pressure–saturation relationship.

Commonly, the capillary pressure–saturation relationship is obtained from direct measurements of capillary pressure and saturation on samples of a material. That relationship strongly depends on the state-of-stress of the sample, among other factors. For example, experiments on soil samples reveal that the air entry pressure increases when the porosity is reduced (Gallipoli et al. 2003; Lins and Schanz 2005; Nuth and Laloui 2008; Tarantino 2009Mašín 2010; Salager et al. 2010; Rostami et al. 2013; Oh and Lu 2014). In order to study the hydromechanical coupling, Salager et al. (2010) performed a large number of drying experiments on clayey silty sand. They showed that there is a relationship among capillary pressure, saturation, and porosity, forming a three-dimensional surface. For swelling absorbing media, the capillary pressure–saturation relationship not only depends on the porosity, but also on the amount of absorbed water (Diersch et al. 2010).

Ideally, for the modelling of hydromechanical coupling, one needs to determine the capillary pressure–saturation relationships for many different packings of a given material under a large number of different stress conditions. This means that many complicated and time-consuming measurements are needed for a complete characterization of a swelling and deformable porous medium. An alternative method is to use a pore-scale model, in combination with limited experimental work, to construct capillary pressure–saturation curves, under a very wide range of conditions. Using pore-scale models, the effect of pore-scale processes on the larger-scale behaviour can be investigated. A commonly used pore-scale model is the pore-network model, where pore bodies are connected by pore throats in a three-dimensional network. For example, Oren et al. (1998) have used a pore-network model to construct the capillary pressure–saturation curve, permeability, and the relative permeability for Bentheimer sandstone, based on a geometry obtained from three-dimensional visualization. Joekar-Niasar et al. (2010) have studied the effect of different pore throat shapes on the capillary pressure–saturation curve for glass-bead packings, using a pore-network model. Raoof and Hassanizadeh (2012) successfully simulated capillary pressure–saturation and relative permeability curves for a carbonate rock sample as well as Fontainebleau sandstone. They included the effect of corner flow in their model. However, pore-network models do not take grain-to-grain interactions into account and do not model grain movements. Therefore, pore-network models alone are not enough for modelling deformation of granular materials.

For pore-scale simulations of a deforming bed of particles, the Discrete Element Method (DEM) is a good alternative. DEM efficiently describes the movement of solid grains inside particle packings during deformation. DEM was introduced by Cundall and Strack (1979). Since then, it has been used for geotechnical simulations of deforming soils (e.g. Widuliński et al. 2009; Belheine et al. 2009; Shmulevich et al. 2007; Wang and Tonon 2010; Plassiard et al. 2009) and particle flow in silos (e.g. Coetzee and Els 2009; Xu et al. 2002; Sykut et al. 2008).

Much research has been conducted on the inclusion of hydromechanical coupling in DEM. Chareyre et al. (2012) have implemented the hydromechanical coupling for saturated conditions. A two-way coupling was presented in Catalano et al. (2014), where fluid exerted forces on the solid grains and the movement of grains influenced the flow of fluid inside the packing. For unsaturated granular materials, Scholtès et al. (2009) have implemented the effect of pendular bridges. However, the pendular bridges were assigned a priori to particles contact area rather than obtained as a consequence of flow of water. Jain and Juanes (2009) developed a two-phase flow algorithm coupled with a DEM model, to study gas migration in sediments. The effect of entry pressure and fracturing of bonds during the process of drainage were considered. Behseresht et al. (2008) coupled the level set method and the DEM model, to simulate the gas–brine interface through a sediment bed. In this way, the mechanistic behaviour of the grains during drainage could be studied. Mousavi and Bryant (2012) have studied the effect of cementation on the capillary pressure saturation curve. They first constructed a random packing of spheres in which the radii of the spheres were increased to simulate cementation, and then, the capillary pressure–saturation curve was constructed by applying a Delauney tessellation, following Behseresht et al. (2008). Gladkikh and Bryant (2005) developed a quasi-static imbibition model that they have applied on a Finney packing (Finney 1970), which is a packing of equally sized spheres of which the coordinates have been measured. Kharaghani et al. (2011) coupled a capillary tube network model and DEM. The capillary tube network simulated time-dependent drying of an initially saturated porous material. By coupling it to DEM, intergranular forces were updated using DEM, depending on the location of the air–water interface. Holtzman and Juanes (2010) have studied two-phase flow patterns in a deformable solid for varying capillary numbers, using a two-dimensional spring dash-pot model. Hydromechanical coupling was achieved by accounting for fluid pressure, which exerts force on a particle, and the contact forces between particles. All aforementioned methods have their merits and disadvantages, but it is clear that insight in hydromechanical coupling can be obtained using DEM. However, the effects of porosity change and swelling of particles on capillary pressure–saturation curves have not yet been studied using the hydromechanical coupling in DEM.

The aim of this research is to study the effects of porosity change and swelling on the capillary pressure–saturation curve, using a pore-scale model. For this purpose, a model has been set up where we have combined a pore-unit assembly method with DEM, to account for hydromechanical coupling. Using this model, we can construct capillary pressure–saturation curves for drainage and imbibition, under a variety of conditions, as described below.

The study material is Absorbent Gelling Material (AGM) particles, which are used in liquid-absorbent hygienic products. AGM particles are capable of absorbing water up to 200 times their initial weight and brine up to 30 times their initial weight (Brandt et al. 1987; Diersch et al. 2010). The hydraulic properties of a bed of AGM particles depend on the induced porosity and/or the amount of absorbed water.

We must point out that we implement a one-way coupling, where the packing does not change during drainage and imbibition. Thus, we assume that imbibition occurs much faster than the absorption of water by the AGM particles and that the imbibition event itself does not rearrange AGM particles. Therefore, we can compute the quasi-static capillary pressure–saturation curve for imbibition in “frozen” packings of spherical particles.

The content is ordered as follows. First, we describe DEM and the pore-unit assembly method. Next, we explain the numerical simulations. Finally, we analyse and discuss the results based on the constructed capillary pressure–saturation curves.

## Numerical Model

### Discrete Element Method

In this research, the open-source software Yade-DEM is used to generate different particle packings. Yade-DEM is based on the Discrete Element Method (DEM) Šmilauer et al. (2015). DEM simulates the movement of individual particles inside a packing made of a large number of particles (Cundall and Strack 1979). For each and every particle, a force balance is written, accounting for the effects of gravity, boundary conditions, and the contact mechanics between particles. Each particle has its own mechanical properties such as Young’s modulus (*E*), Poisson ratio $(\nu )$, and friction angle $(\varphi )$ as well as a radius (*R*) and density.

Regarding the contact mechanics between two particles, consider a particle *i* in contact with a neighbouring particle *j*. Particles *i* and *j* are pushed towards each other due to their surrounding particles. This causes a relative movement of particle *i* towards particle *j* and consequently the flattening of both particles at the contact. The tendency of a particle to maintain its original spherical shape causes an elastic force at the contact (Johnson and Johnson 1987; Popov 2010). The elastic response, in terms of normal force $(f_n)$, is expressed using either a linear function or in our case, a physically based Hertz-Mindlin equation (e.g. Thornton et al. 2011):

1 $$\begin{aligned} f_n =-k_n \delta _n ^{3/2} \end{aligned}$$

where $\delta _n$ is the normal displacement of particles *i* and *j* towards each other, and $k_n$ is the contact stiffness in the normal direction that is given by: $k_n=\frac{4}{3}E^{\mathrm{*}}\sqrt{R^{\mathrm{*}}}$. It depends on the properties of the two interacting particles through: $E^{*}=\left( {\frac{1-\nu _i ^{2}}{E_i}+\frac{1-\nu _j ^{2}}{E_j }} \right) ^{-1}$ and $R^{*}=\frac{R_i R_j }{R_i +R_j }$, where $\nu$, *E*, and *R* are the Poisson ratio, Young’s modulus, and the radius of each particle, respectively.

At the contact point between two particles, not only normal displacement, but also shear may occur. The elastic force in the tangential direction depends on the tangential displacement $(\mathbf{d}_\mathbf{t} )$. The rate of change in tangential force $({\dot{\mathbf{f}}_\mathbf{t}})$ is calculated by:

2 $$\begin{aligned} {{\dot{\mathbf{f}}}_\mathbf{t}}=k_{t}{{\dot{\mathbf{d}}}_\mathbf{t}} \end{aligned}$$

where ${{\dot{\mathbf{d}}}_\mathrm{t}}=\left( {\dot{\mathbf{x}}_\mathbf{i}}- {\dot{\mathbf{x}}_\mathbf{j}}\right) + {\varvec{\upomega }}_{\mathbf{i}}\times (\mathbf{x}_\mathrm{c}-\mathbf{x}_\mathrm{i})-{\varvec{\upomega }}_\mathrm{j}\times (\mathbf{x}_\mathrm{c}-\mathbf{x}_\mathrm{i})$. It is the sum of the relative velocity of the two particles $({\dot{\mathbf{x}}_\mathbf{i}}$ and ${\dot{\mathbf{x}}_\mathbf{j}})$ and their rotation: ${\varvec{\upomega }}_\mathbf{i} \times \left( {\mathbf{x}_\mathrm{c} -\mathbf{x}_\mathrm{i} } \right)$ where ${\varvec{\upomega }}_\mathbf{i}$ is the angular velocity vector of particle *i* and $\mathbf{x}_\mathrm{c}$ is the position vector of the contact point. The contact stiffness in the tangential direction $(k_t)$ is given by (Tsuji et al. 1991; Johnson and Johnson 1987):

3 $$\begin{aligned} k_t =\frac{4\sqrt{R^{{*}}}{\bar{G}}}{2-{\bar{\nu }}}\delta _n ^{0.5} \end{aligned}$$

where ${\bar{G}}$ and ${\bar{\nu }}$ are the arithmetic averages of the shear moduli and the Poisson ratios of the two particles. If the tangential force surpasses a threshold value, sliding occurs. According to the Mohr-Coulomb criteria for static friction, this will happen when the following holds:

4 $$\begin{aligned} |{f}_{t}|= f_n \tan \varphi \end{aligned}$$

where $\varphi$ is the internal friction angle. All contact forces that act on particles are integrated using Newton’s second law, to find the particle motions. For more information, the reader is referred to Šmilauer et al. (2015).

### Pore-Unit Model

#### Extraction of Pore-Unit Assembly

The pore space of a granular medium can be subdivided into pore bodies and pore throats. Pore bodies contain most of the void volume, while pore throats are the relatively narrow transects that connect pore bodies to each other. In case of a packing of spherical particles, the pore space is often extracted using a classic triangulation (Gladkikh and Bryant 2005; Mason and Mellor 1995) or a regular triangulation (Chareyre et al. 2012; Yuan et al. 2015; Catalano et al. 2014). A regular triangulation is capable of subdividing the pore space of packings with variable particle sizes, in contrast to classic triangulation which can only handle constant particle sizes. Here, a regular triangulation is employed to subdivide the pore space into tetrahedra, each formed by four neighbouring particles. Vertices of a tetrahedron are located at the centres of those particles. The pore space enclosed within a tetrahedron is called a pore unit. Thus, the network of tetrahedra forms a pore-unit assembly.

**Fig. 1:**
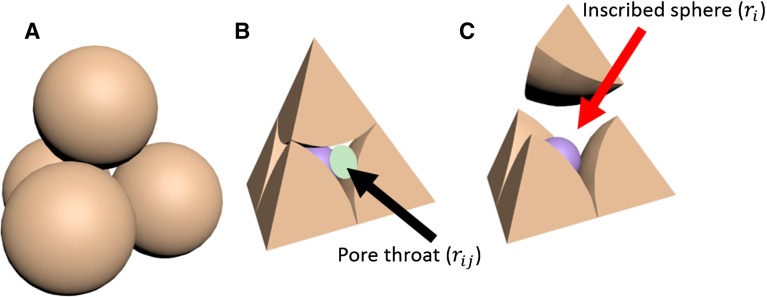
Illustration of a pore unit: **a** A pore unit that is enclosed by four particles. **b** A tetrahedron that encloses one pore unit, where the *green circle* shows the inscribed *circle* of a pore throat. **c** An inscribed *sphere* in a pore unit; note that the upper grain has been moved away to expose the inscribed sphere

The geometry of each individual tetrahedron enclosing one pore unit (see Fig. 1) can be described as follows. (1) Each vertex of the tetrahedron is at the centre of a particle. (2) In each corner of a tetrahedron, a part of a particle is located. (3) One tetrahedron is connected to 4 neighbouring tetrahedra. (4) The facet of a tetrahedron is shared by two touching tetrahedra and is considered as a pore throat.

#### Invasion Criterion

The pore-unit assembly is used to construct the capillary pressure–saturation curves. To do so, two geometrical properties of a pore unit are essential. These are the radius of the inscribed circle of a pore throat $(r_{ij})$ and the radius of the inscribed sphere of a pore unit $(r_i)$, which are shown in Fig. 1. Both properties are used to determine the invasion criteria for drainage and imbibition. For drainage, a single invasion criterion is considered, namely the entry pressure associated with the pore throats. For imbibition, two criteria are considered, namely the entry pressure of a throat, and the largest stable curvature inside a pore unit.

To determine the entry pressure of a pore throat, consider two connected pore units (*i* and *j*) that have a shared pore throat with an inscribed circle radius $$r_{ij}$$. If pore unit *i* is saturated with air and pore unit *j* is saturated with water, then an air–water interface is located at pore throat *ij*. The air–water interface will have a complex shape, resembling both a spherical cap and a liquid bridge. Here, it is assumed that the air–water interface has the shape of a spherical cap, following Gladkikh and Bryant (2005) and Torskaya et al. (2014). Therefore, the entry pressure of a throat, $$P_e^{ij} $$, is given as:

5 $$\begin{aligned} P_e^{ij} =\frac{2\gamma }{r_{ij}} \end{aligned}$$

where $$\gamma $$ is the interfacial surface tension between water and air $$(72.0\,\hbox {dynes cm}^{-2})$$. For air to invade pore *j*, the pressure difference between the air and water reservoir $$(P^{i}-P^{j})$$ should be larger than the entry pressure $$(P^{i}-P^{j}>P_e^{ij})$$. This is the criterion used during drainage. We assume that once the pore throat *ij* is invaded, the pore unit *j* will be instantaneously filled by the air phase. New air–water interfaces will be formed at pore throats between pore unit *j* and its neighbouring pore units. Criterion $$P^{i}-P^{j}>P_e^{ij} $$ will be checked again.

For imbibition, consider pore units *j* and *i* filled by water and air, respectively. For water to invade the air-saturated pore unit *i* via pore throat *ij*, the pressure difference $P^{i}-P^{j}$ should be lower than the entry pressure $P_e^{ij}$. But, pore unit *i* will not be fully invaded by water. Stable air–water interfaces can form within the pore unit. For full invasion of the pore unit to occur, the pressure difference should drop below a threshold value. This threshold value is equal to $\frac{2\gamma }{r_i}$, where $r_i$ is the largest radius of curvature of a stable air–water interface that can exist inside a pore unit, which is that of the inscribed sphere of pore unit *i*. The radius of the inscribed sphere $(r_i)$ is found using a Cayley–Menger determinant, which is a method for finding the radius of an inscribed sphere for four neighbouring spheres (whether touching or nontouching). For more information, the reader is referred to MacKay (1973) and Michelucci and Foufou (2004).

A complication occurs when a cluster of particles is not closely packed and, as a result, those particles form a large pore wherein more than one inscribed sphere can be fitted. In this situation, triangulation may subdivide a large pore into two or more tetrahedra, even though it should be considered as one single pore. Then, it is possible that these pore units are separated by a “pore throat” whose radius $$r_{ij}$$ is larger than the radii of the inscribed spheres. This is merely an artefact of triangulation. The presence of larger pores does not affect the simulations of drainage, since the smallest pores determine the location of the air–water interface. For imbibition, we allow $$r_{ij} $$ to be larger than $$r_i $$ since the largest hemisphere that we can fit in a pore should determine the threshold pressure for imbibition.

**Fig. 2:**
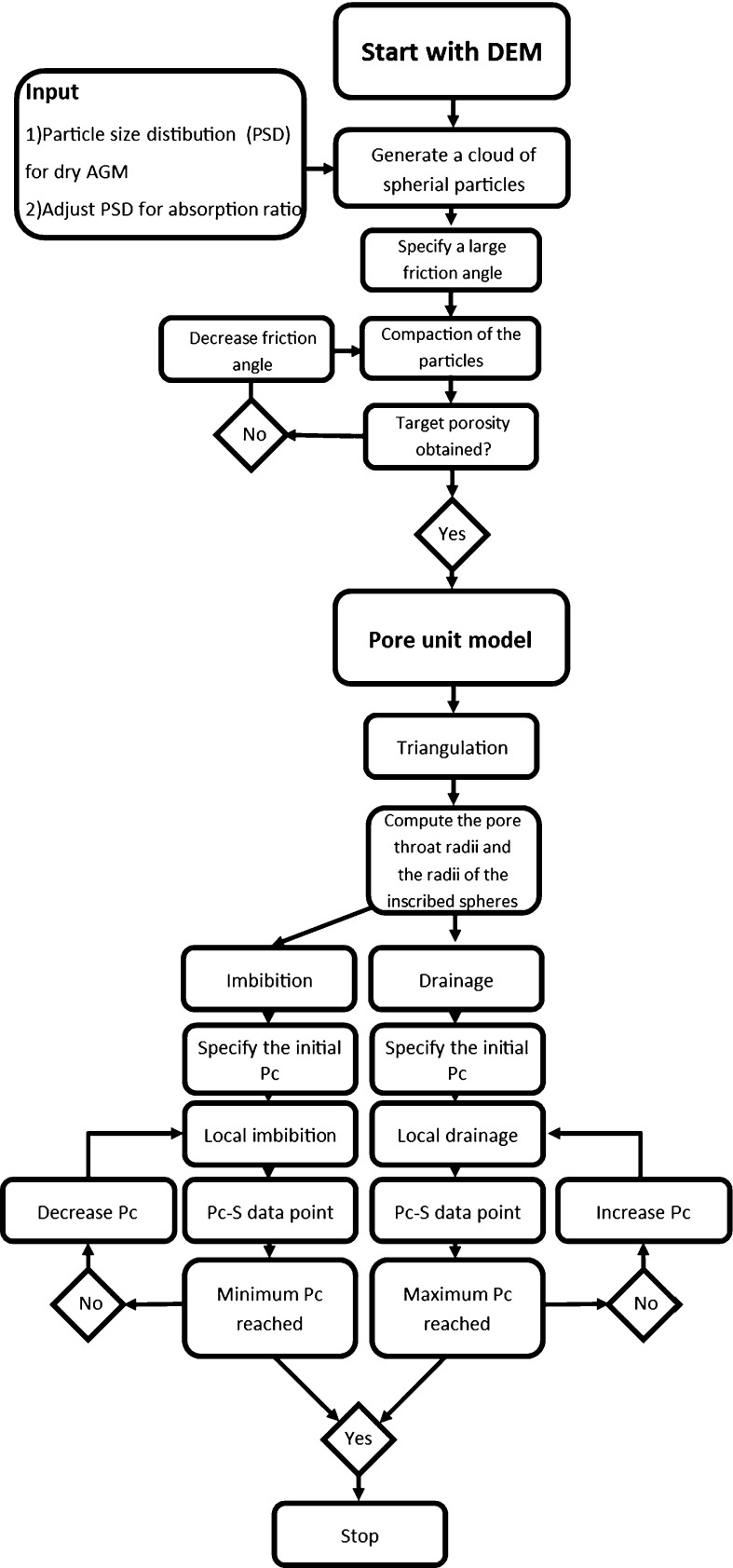
Flow chart for the calculation of capillary pressure–saturation curves

## Numerical Simulations

The general outline of the numerical simulations is shown in a flow chart in Fig. 2. First, for a certain amount of absorbed water and porosity, we generate a particle packing using Yade-DEM. For this particle packing, the pore geometry is extracted using the pore-unit assembly method. Finally, the capillary pressure–saturation curve is constructed. The procedure is explained in detail in the following sections.

### The Particle Size Distribution and the Water Absorption Ratio

In our simulations, we start with a given particle size distribution that corresponds to that of dry AGM particles. This is then adjusted to account for the target value of the amount of absorbed water. The amount of absorbed water is quantified using the absorption ratio (*Q*), as defined by Buchholz and Graham (1998):

6 $$\begin{aligned} Q=\frac{M_w +M_s}{M_s} \end{aligned}$$

where $$M_s$$ is the mass of dry AGM and $$M_w $$ the mass of absorbed water. As the particles are assumed to be spherical, we may recast Eq. (6) into:

7 $$\begin{aligned} Q=\frac{M_w +M_s}{M_s}=\frac{\rho _w \left[ {\left( {R_i } \right) ^{3}-\left( {R_i^0 } \right) ^{3}} \right] +\rho _s \left( {R_i^0 } \right) ^{3}}{\rho _s \left( {R_i^0 } \right) ^{3}} \end{aligned}$$

where $$R_i^0 $$ and $$R_i $$ are the radii of particle *i* before and after swelling, respectively. $$\rho _w $$ is the density of water, which is set to $$1\,\hbox {g cm}^{-3}$$ and $$\rho _s $$ is the density of dry AGM, which is set to $$1.6\,\hbox {g cm}^{-3}$$, following the reported value by Mirnyy et al. (2012). Equation 7 is inverted to obtain:

8 $$\begin{aligned} R_i=R_i^0 \root 3 \of {1+\frac{\rho _s }{\rho _w }\left( {Q-1} \right) } \end{aligned}$$

Thus, an initial particle size distribution with radii $$R_i^0 $$ can now be adjusted for the amount of absorbed water. The particle packing is then created with this size distribution.

### Generating Particle Packings

The particle packings were generated for a predefined particle size distribution corresponding to a given absorption ratio, and for a target porosity. The dry AGM particle radii were assumed to have a mean value of $150\,\upmu \hbox {m}$ and a normal distribution with a standard deviation of $30\,\upmu \hbox {m}$. First, a cloud of particles was generated within a cubic domain with dimensions 0.15 m $\times 0.15$ m $\times 0.15$ m, using the particle size distribution with radii scaled for the absorption ratio according to Eq. (8). The Poisson ratio of the particles was set to 0.5, because swollen AGM particles contain predominantly water and therefore we assume the particles to be incompressible. The Young’s modulus of the particles was set to 100 kPa. This value corresponds to slightly swollen AGM particles (see for example Knaebel et al. (1997)). Next, the cloud of particles was compacted by applying a confining pressure, which was different for different target porosities (50 Pa for a target porosity of 0.45 and 2.500 Pa for a target porosity of 0.10). For this step in the simulations, we started with an artificially large friction angle of $50^{\circ }$. This was done to obtain a relatively loose packing with a larger porosity than the target porosity. Then, to reduce the porosity, the contact friction was reduced progressively such that the particles would slide into a more stable configuration, until the porosity matched the target porosity. This method is based on that of Chareyre et al. (2002). The final particle packing was at equilibrium under the applied confining pressure.

### Modelling the Capillary Pressure–Saturation Curve

To obtain the capillary pressure–saturation curve, the boundary conditions in the pore-unit model were chosen to mimic a column experiment. The top boundary of the modelling domain in Yade-DEM was assumed to be a nonwetting phase reservoir (e.g. air) at a pressure $P_{nw}$; the bottom boundary was considered to be a wetting phase reservoir (e.g. water) at a pressure $P_w$. For quasi-static simulations, where the viscous effect of water flow is not of importance, the capillary pressure $(P_c )$ is equal to the pressure difference between the two reservoirs:

9 $$\begin{aligned} P_c =P_{{ nw}}-P_w \end{aligned}$$

For drainage simulations, we started with a very low capillary pressure, which was then increased incrementally in order to allow air to infiltrate increasingly smaller pores. For imbibition simulations, we started with a very large capillary pressure, which was then decreased incrementally such that water could invade increasingly larger pores.

In the pore-unit model, the simulation procedure was as follows: (1) a capillary pressure was specified. (2) For drainage, the pore throats of all water-saturated pore units were checked to determine whether they could be invaded by air. For imbibition, the pore throats of all air-saturated pore units were checked whether water could invade. (3) Drainage of a water-saturated pore unit by air (or vice versa during imbibition) was allowed only when both phases were connected to their corresponding reservoirs. (4) Capillary pressure was increased during drainage and decreased during imbibition. (5) An algorithm was implemented to keep track of disconnected blobs of air and water, to ensure that no displacement of disconnected blobs could occur. These disconnected blobs would form the residual air or water saturation at the end of either imbibition or drainage, respectively.

### Van Genuchten Function

All simulated capillary pressure–saturation points were fitted with the Van Genuchten function (Van Genuchten 1980):

10 $$\begin{aligned} S_e =\frac{1}{\left( {1+\left| {\alpha P_c } \right| ^{n}} \right) ^{1-1/n}} \end{aligned}$$

where $$\alpha \,[\hbox {Pa}^{-1}]$$ and *n* are fitting parameters, representing the inverse of the entry pressure and the pore size distribution, respectively, and $$S_e $$ is the effective saturation defined as:

11 $$\begin{aligned} S_e =\frac{S-S_r}{S_{\mathrm{max}} -S_r} \end{aligned}$$

Here, *S* is the saturation, $$S_r$$ is the residual water saturation after drainage, and $$S_{\mathrm{max}}$$ is the maximum water saturation after imbibition. For results on primary imbibition $$S_r $$ was set to 0.0. Drainage and imbibition curves were separately fitted with the Van Genuchten function, leading to separate Van Genuchten parameters for drainage and imbibition, namely: $$\alpha _{\mathrm{dr}} $$, $$\alpha _{\mathrm{imb}}$$, $$n_{\mathrm{dr}}$$, $$\alpha _{\mathrm{imb}}$$, where subscript dr indicates drainage, and imb indicates imbibition. All results were fitted using SWRC fitting program (Seki 2007).

## Results

### Model Testing

In this section, we test our pore-unit model by constructing capillary pressure–saturation curves for Hostun sand, for which the measured drainage and imbibition curves were found in the literature. Hostun sand was chosen instead of AGM particles, because only few data are available on the capillary pressure–saturation curves for AGM particle beds. We generated a packing of Hostun sand, based on the particle size distribution and a porosity of 0.39 as reported by Lins and Schanz (2005).

**Fig. 3:**
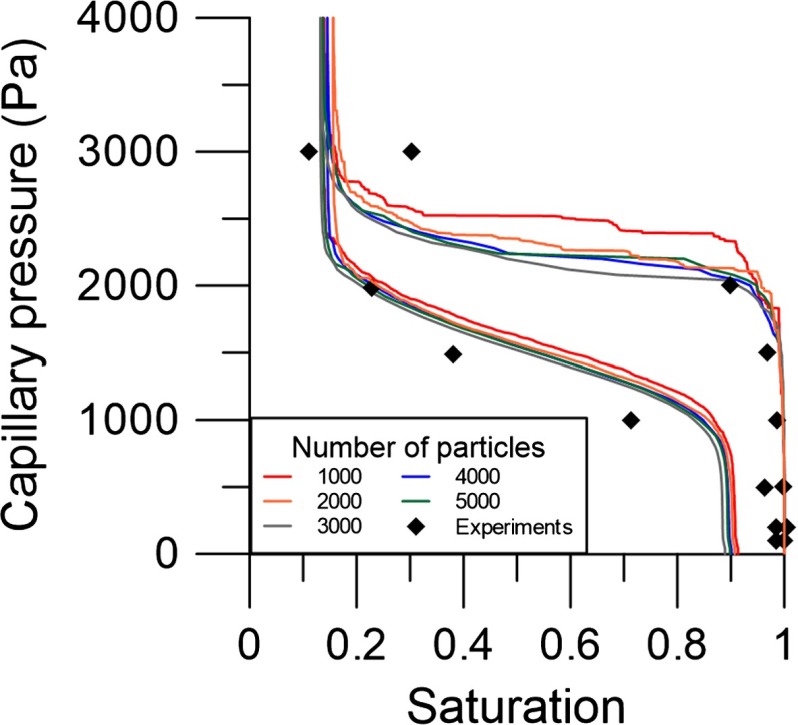
The capillary pressure–saturation curves for Hostun sand with a porosity of 0.39, for packings of different number of particles (*solid lines* of different colours). *Symbols* show experimental data by Lins and Schanz (2005) for Hostun sand

First, the simulations for Hostun sand were used to determine the minimum number of particles that is required for the capillary pressure–saturation curve to be independent of the number of particles. This follows the concept of Representative Elementary Volume (REV), which implies that the effects of small-scale heterogeneities are averaged out. The calculations were done for packings with different numbers of particles, varying from 1000 to 5000. Results showed that different primary drainage curves were obtained for different numbers of particles (see Fig. 3). But, the curves converged for packings of 4000 particles and more. The main imbibition curves were less sensitive to the number of particles of the packing. Packings of 4000 particles have approximately 19,000 pore units and 38,000 pore throats, not including the boundary pore units.

**Fig. 4:**
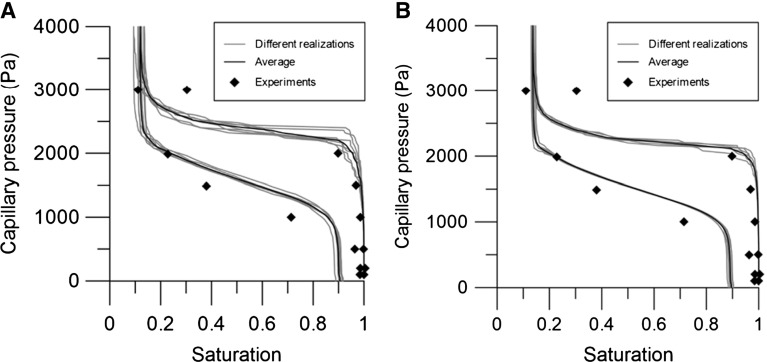
The capillary pressure–saturation curves (*thin lines*) for Hostun sand with a porosity of 0.39, for different realizations of the packing: **a** 1000 particles, **b** 4000 particles. The average curve is shown by the *thick line*. Experimental data by Lins and Schanz (2005)

Another aspect related to the concept of REV is the variation in simulated curves for different realizations of the same packing. To illustrate this variation, different packings with the same number of particles were generated for different random distributions in the particle location. This was done for two cases: 1000 and 4000 particles. For each case, six capillary pressure–saturation curves were constructed. Figure 4a shows that the primary drainage curves obtained for different realizations of a packing of 1000 particles were substantially different from each other. However, for 4000 particles, there was much less variation as shown in Fig. 4b. For both 1000 and 4000 particles, the main imbibition curve varied very little for different realizations.

Based on these results, we decided that the REV size should be around 4000 particles. All simulations hereafter were carried out for an assembly of 4000 particles.

First, we simulated capillary pressure–saturation curves for Hostun sand and compared them to experimental data by Lins and Schanz (2005), as shown in Fig. 4b. Lins and Schanz (2005) measured the capillary pressure–saturation curve for capillary pressures up to 80 kPa. But, for capillary pressures higher than 4kPa water was found to be almost in a residual state. Water saturation decreased only when the capillary pressure was increased substantially; this process was not included in our model. Therefore, the experimental data by Lins and Schanz (2005) are shown here for capillary pressures up to 4 kPa. Figure 4b shows that our simulations of Hostun sand are in reasonable agreement with the measured curve. We have fitted the experimental data and the simulated curves with the Van Genuchten function, and values of $\alpha$ and *n* are shown in Table 1. Results show that the values of *n* for drainage $(n_{\mathrm{dr}})$ and imbibition $(n_{\mathrm{imb}})$ are larger for the simulated curves than for experimental data, because the simulated curves are less steep than the experimental data. The value of $\alpha$ for drainage $(\alpha _{\mathrm{dr}})$ is overestimated by the simulated curves compared to experimental data, whereas the value of $\alpha$ for imbibition $(\alpha _{\mathrm{imb}})$ is underestimated.

**Table 1:** Van Genuchten parameters for drainage and imbibition capillary pressure–saturation curves for the experimental data by Lins and Schanz (2005) and the simulated curves for 4000 particles

	Experimental data	Simulated curves
$\alpha _{\mathrm{imb}}$ (1/Pa)	$8.76\times 10^{-4}$	$6.8\times 10^{-4}$
$n_{\mathrm{imb}}$	3.8	6.6
$\alpha _{\mathrm{dr}}$ (1/Pa)	$3.95\times 10^{-4}$	$4.5\times 10^{-4}$
$n_{\mathrm{dr}}$	7.9	21

The residual water saturation in our simulations was 0.14, which was close to the measured value of 0.11. Due to the angular shape of sand grains, corner flow of water at low saturation allows for additional drainage, and therefore, a lower saturation at higher capillary pressure can be reached. Moreover, the maximum water saturation after imbibition was 0.90, which is lower than the experimental value, namely 0.98. This may be related to the fact that particles are modelled as spheres in DEM, whereas sand is typically less round and more angular. The maximum water saturation is lower in packings of spherical grains. For example, the maximum water saturation in experiments with glass-bead packings has been found to be 0.92 (Culligan et al. (2004)), or 0.74–0.92 (Dullien et al. 1989).

### Capillary Pressure–Saturation Relationship of AGM Particles Beds

Capillary pressure–saturation curves for main drainage and primary imbibition were simulated for a typical range of absorption ratios: from 5 to 40 g/g (cf. Brandt et al. 1987) and porosity values: from 0.10 to 0.43 (cf. Mirnyy et al. 2012). In this section, we report the dependency of the capillary pressure–saturation curve on porosity and absorption ratio.

#### Effects of Porosity and Absorption Ratio

Diersch et al. (2010) have developed a macro-scale numerical model for swelling granular materials under partially saturated conditions. In that model, the Van Genuchten parameter $\alpha$ was considered to be dependent on the absorption ratio (*Q*) and the porosity $(\phi )$, based on experimental evidence. But, Van Genuchten parameter *n* was assumed to be constant. In their model, the load that was applied (*P*) to confine the sample was assumed to be constant. Therefore, the porosity was only considered to change with changing absorption ratio. Thus, it was assumed that both $\alpha$ and $\phi$ uniquely vary with *Q*. Both dependencies were experimentally determined and used as input for the macro-scale simulations. However, for a varying confining load, porosity can change independently of *Q*. Thus, $\alpha$ should be considered to be dependent on porosity and absorption ratio. To study the effects of both $\phi$ and *Q* on $\alpha$ separately, we kept one variable constant while varying the other. By keeping *Q* constant while varying $\phi$, the effect of consolidation on $\alpha$ was studied. On the other hand, we increased *Q* while releasing net stress in order to keep $\phi$ constant.

**Fig. 5:**
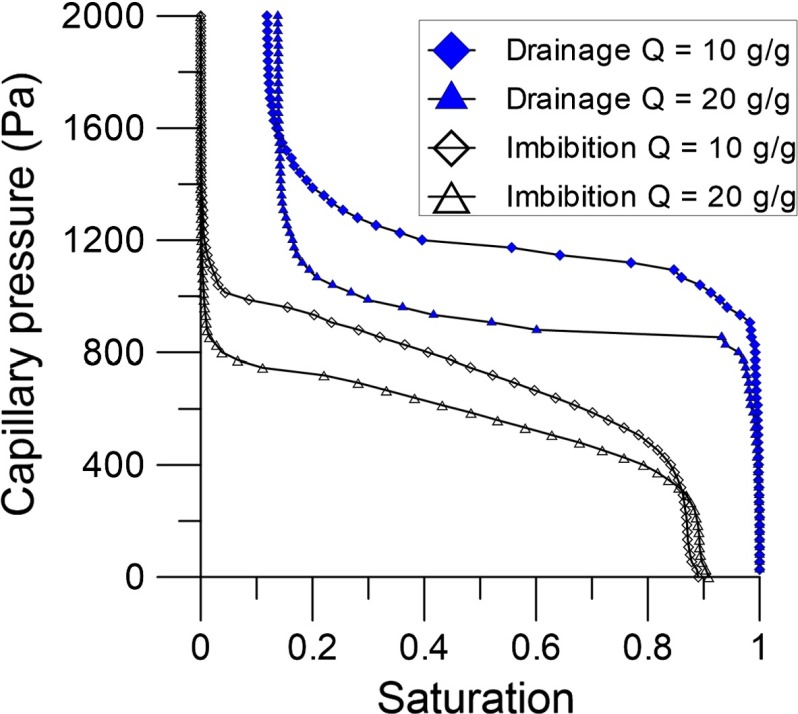
Drainage and imbibition capillary pressure–saturation curves for different values of absorption ratio at a constant porosity of 0.30

**Fig. 6:**
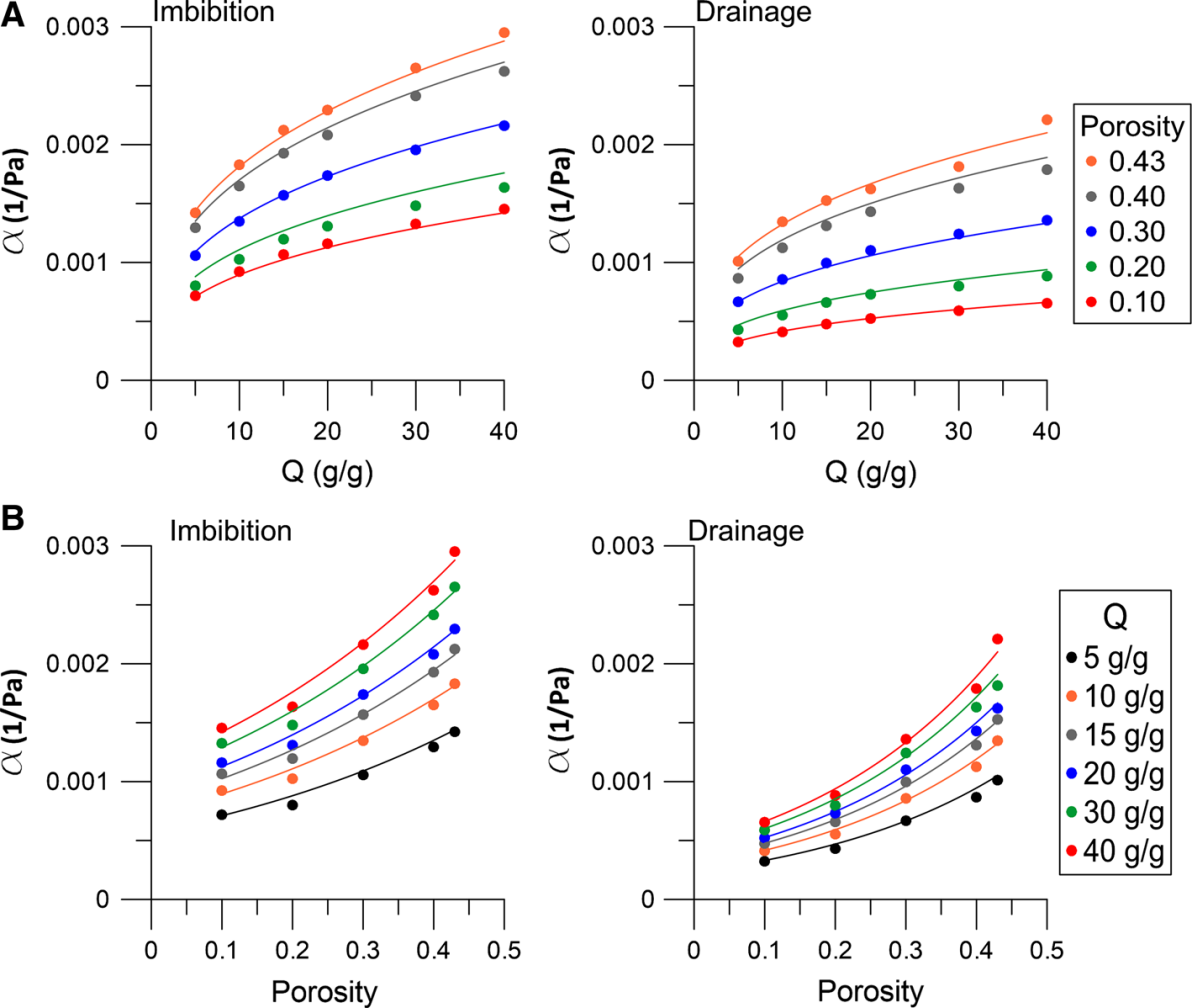
Van Genuchten parameter $\upalpha$ for imbibition and drainage as a function of **a** the absorption ratio and **b** the porosity. *Dots* represent values of $\upalpha$ of simulated capillary pressure–saturation curves, while *coloured lines* represent *fitting curves* that are given in Eq. (12)

First, capillary pressure–saturation curves were obtained for different absorption ratios, at a variety of constant porosity values. Results are shown in Fig. 5. These curves were fitted with the Van Genuchten formula $({R}^{2} >0.98)$ such that the values of $\alpha _{\mathrm{dr}}$, $n_{\mathrm{dr}}$, $\alpha _{\mathrm{imb}}$, and $n_{\mathrm{imb}}$ were determined. In general, increasing the absorption ratio at a fixed porosity causes the volume of the sample to increase (due to larger grain sizes). Packings with larger grains, at a constant porosity, will have a lower entry pressure (and thus a higher value of $\alpha _{\mathrm{dr}}$ and $\alpha _{\mathrm{imb}}$), because the pore sizes become larger. Indeed, our results showed that both $\alpha _{\mathrm{dr}}$ and $\alpha _{\mathrm{imb}}$ increase with an increasing *Q*, as shown in Fig. 6a.

Next, capillary pressure saturation curves were obtained for packings with different porosities, at a number of constant absorption ratios. Results show that both $\alpha _{\mathrm{dr}}$ and $\alpha _{\mathrm{imb}}$ decrease (i.e. we will have a larger entry pressure) with decreasing porosity as seen in Fig. 6b. An increase in entry pressure, due to a decrease in porosity is in agreement with experimental observations that are reported in the literature (Vanapalli et al. 1996; Lins and Schanz 2005). For example, Lins and Schanz (2005) have shown that the entry pressure for imbibition increases from 0.3 kPa for loosely packed Hostun sand with a porosity of 0.47 to 0.7 kPa for densely packed Hostun sand with a porosity of 0.39. This corresponds to a change in $\alpha$ from $0.0033\,\hbox {Pa}^{-1}$ to 0.0014. Oh and Lu (2014) studied the retention properties of weathered granite. They found an increase in entry pressure, for main imbibition, from 1130 to 2110 Pa for a change in porosity from 0.37 to 0.32. This corresponds to a change in $\alpha$ from $0.0009\,\hbox {Pa}^{-1}$ to 0.0005. However, they noted that the main imbibition curves were less affected by a change in porosity than the drainage curves.

**Fig. 7:**
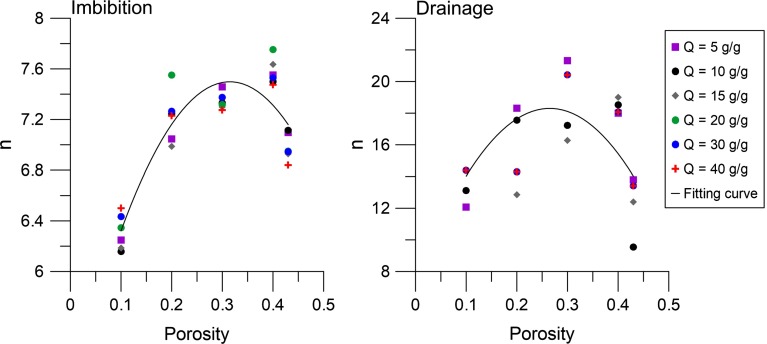
The Van Genuchten parameter *n* as a function of porosity for imbibition and drainage. *Dots* represent data points from simulated capillary pressure–saturation curves, while *solid lines* represent *fitting curves* that are given by Eqs. (13) and (14) for imbibition and drainage, respectively

The results in Fig. 6a, b were fitted by the following formula $(R^{2}>0.98)$:

12 $$\begin{aligned} \alpha _{\mathrm{dr}}= & {} e^{3.50\phi -8.9}\times Q^{1/3} \nonumber \\ \alpha _{\mathrm{imb}}= & {} e^{2.14\phi -8.0}\times Q^{1/3} \end{aligned}$$

Note that Eq. (12) is valid for porosity values varying between 0.10 and 0.43. Diersch et al. (2011) assumed the value of $\alpha _{\mathrm{dr}}$ to be $\frac{1}{2}\alpha _{\mathrm{imb}}$. That ratio falls within the range of values suggested by Eq. (12). There we find that $\alpha _{\mathrm{dr}}$ varies from $0.40\alpha _{\mathrm{imb}}$ (for $\phi =0.10$) to $0.72\alpha _{\mathrm{imb}}$ (for $\phi =0.43$).

Moreover, we found that the Van Genuchten parameter *n* has a weak dependency on the porosity value and has no dependency on the absorption ratio (see Fig. 7). The value of *n* during imbibition $(n_{\mathrm{imb}})$ was fitted with a second-order polynomial equation $({R}^{2} = 0.82)$:

13 $$\begin{aligned} n_{\mathrm{imb}} =-25.5\phi ^{2}+16.1\phi +5.0, \end{aligned}$$

whereas for drainage the values of *n* were much more scattered and as a consequence the quality of the fitting was less $({R}^{2}=0.32)$. Due to the large values of $n_{\mathrm{dr}}$, namely $n_{\mathrm{dr}} >12$, small difference in $n_{\mathrm{dr}}$ does not affect the capillary pressure–saturation curve significantly. Therefore, the capillary pressure–saturation curves can be relatively close to each other, but can have large variation in $n_{\mathrm{dr}}$. The fitting equation is given by:

14 $$\begin{aligned} n_{\mathrm{dr}} =-157\phi ^{2}+83.3\phi +7.3 \end{aligned}$$

**Fig. 8:**
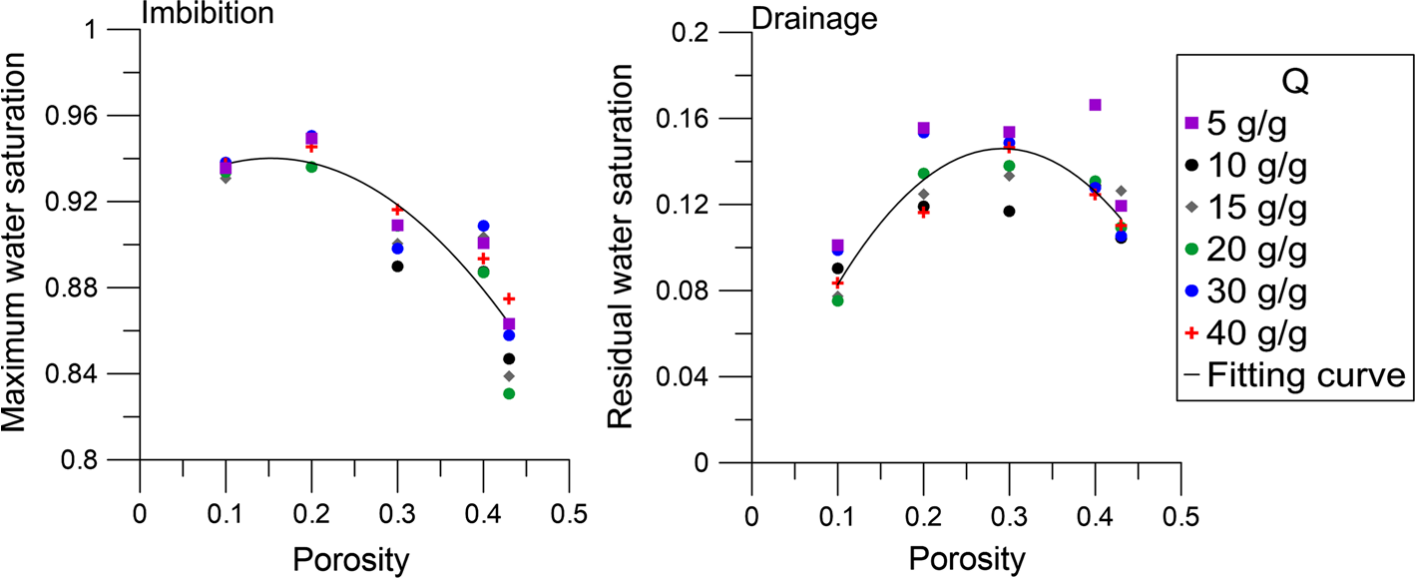
Maximum water saturation during imbibition $(S_{\mathrm{max}})$ and residual water saturation during drainage $(S_r^w)$ as a function of porosity. *Dots* represent data points from simulated capillary pressure–saturation curves, while *solid lines* represent fitting curves that are given by Eqs. (16) and (17)

**Fig. 9:**
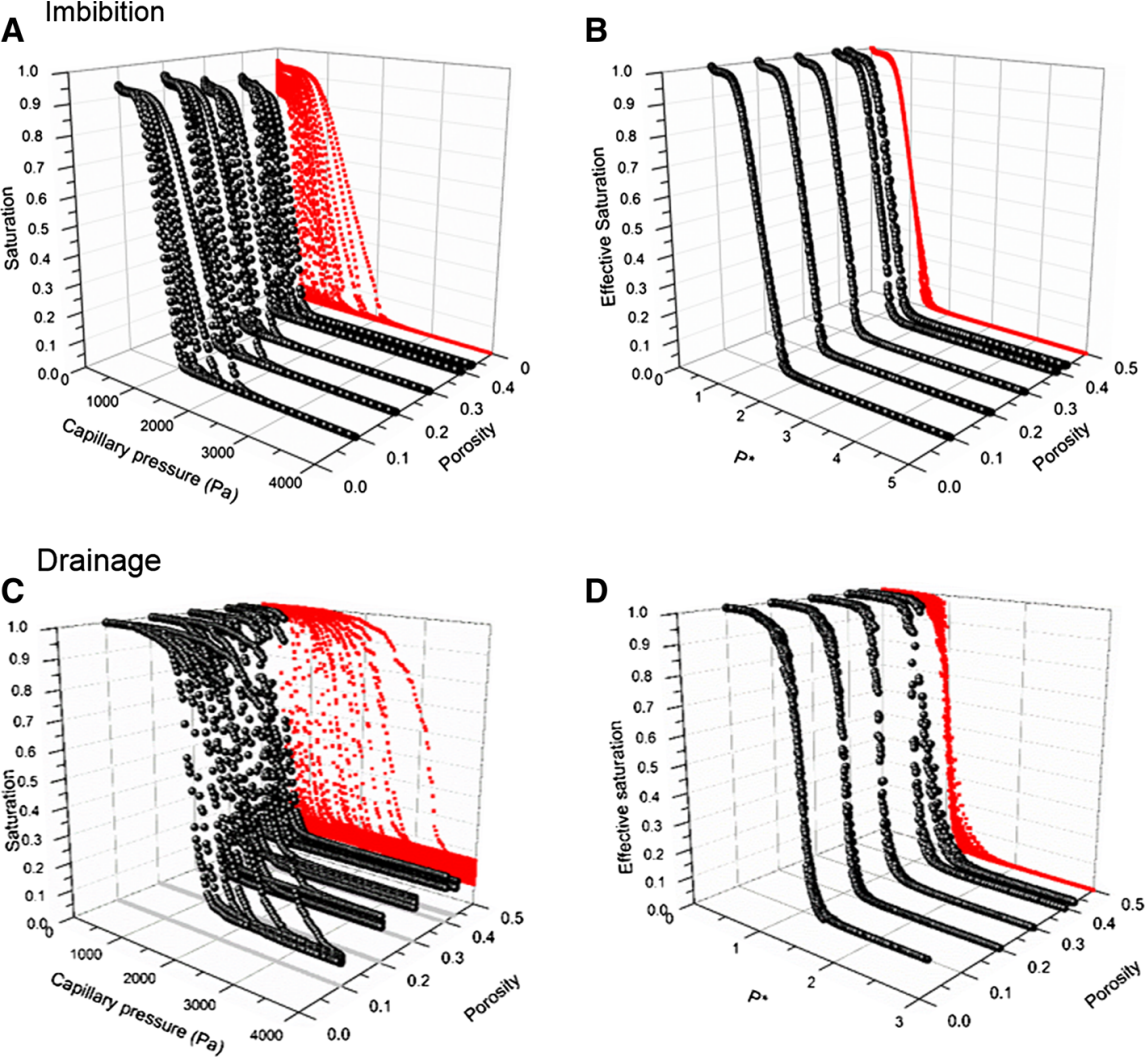
${P}_\mathrm{c}$–*S*–$\upphi$ *plots* for AGM particles at different absorption ratios. For each porosity value, there are five different ${P}_\mathrm{c}$–*S* curves corresponding to different values of absorption ratios. *Red* curves show the projections of ${P}_\mathrm{c}$–*S* curves on the ${P}_\mathrm{c}$–*S* plane. **a** All imbibition capillary pressure–saturation curves of AGM particles. **b** *Plot* of the normalized capillary pressure $({P}^{\mathrm{*}}={P}_\mathrm{c}\upalpha )$ versus effective saturation $({S}_{\mathrm{eff}})$ for imbibition. **c** All drainage capillary pressure–saturation curves of AGM particles. **d** *Plot* of the normalized capillary pressure $(\hbox {P}^{\mathrm{*}}={P}_\mathrm{c} \upalpha )$ versus effective saturation $({S}_{\mathrm{eff}})$ for drainage

#### Maximum Water Saturation

Another aspect of the capillary pressure–saturation curve is the amount of trapped air after primary imbibition. In our model, residual air is present as disconnected air-saturated pore units. A trapped air ganglion is formed when the movement of water disconnects a cluster of air-saturated pore units from the air reservoir. The residual air saturation formed at the end of an imbibition process $(S_r^{\mathrm{air}})$ corresponds to the maximum water saturation $(S_{\mathrm{max}})$:

15 $$\begin{aligned} S_r^{\mathrm{air}} =1-S_{\mathrm{max}} \end{aligned}$$

The residual air saturation depends on the pore structure of the porous medium. Figure 8 shows that the maximum water saturation (or the residual air saturation) depends on the porosity of the packing but not much on the absorption ratio. The absorption ratio causes swelling of all particles and thus does not change the network of pore units other than a translation of the size distribution. Therefore, the maximum water saturation is almost insensitive to the absorption ratio.

However, the maximum water saturation does depend on the porosity; the higher the porosity, the lower the maximum water saturation. This phenomenon can be explained by the micro-scale heterogeneity of the pore structure. At higher porosity values, more clusters exist that contain relatively larger pore units. Water will flow via the smaller surrounding pore units rather than the high-porosity clusters, leading to the trapping of residual air phase in the high- porosity clusters. The maximum saturation was fitted as function of porosity $(R^{2} = 0.78)$, but $S_{\mathrm{max}}$ was not dependent on *Q*, such that $S_{\mathrm{max}}$ is described by:

16 $$\begin{aligned} S_{\mathrm{max}} =-\phi ^{2}+0.31 \phi +0.92 \end{aligned}$$

Equation (16) is valid for porosity values between 0.10 and 0.43.

#### Residual Water Saturation

Another feature of the capillary pressure–saturation curve for main drainage is the residual water saturation $$(S_r^w )$$. Figure 8 shows a weak dependency of residual water saturation on the porosity. We have fitted the $$S_r^w $$ on porosity using a second-order polynomial equation $$(R^{2}=0.67)$$ for porosity values varying from 0.10 to 0.43:

17 $$\begin{aligned} S_r^w =-1.7\phi ^{2} + \phi \end{aligned}$$

#### Normalizing the Capillary Pressure–Saturation–Porosity–Absorption Data Points

We have plotted the capillary pressure–saturation curves for drainage and imbibition, for different porosity values and absorption ratios, with the following axes: capillary pressure, saturation, and porosity (see Fig. 9a, c). The two-dimensional projection of the resulting three-dimensional surface on the capillary pressure–saturation plane shows the spreading in the capillary pressure–saturation curves for both the drainage and imbibition. We used Van Genuchten parameter $\alpha$ for each capillary pressure–saturation curve to normalize the capillary pressure by defining:

18 $$\begin{aligned} P^{*}= P_c \alpha \end{aligned}$$

We also replaced saturation values for each curve with the effective saturation. The plots of $P^{*}$ versus effective saturation are shown in Fig. 9b, d. As can be seen, the curves for drainage have collapsed into one single curve for drainage and the curves for imbibition have collapsed into one single curve for imbibition, showing that $P^{*}\left( {S_{\mathrm{eff}} } \right)$ is independent of the porosity value and the absorption ratio. The value of $n_{\mathrm{imb}}$ for the $P^{*}\left( {S_{\mathrm{eff}} } \right)$ relationship for imbibition was 7.03, which is close to the average value of $n_{\mathrm{imb}}$ for all individual drainage curves, namely 7.24. Moreover, the value of $n_{\mathrm{dr}}$ for the $P^{*}\left( {S_{\mathrm{eff}}}\right)$ relationship for drainage was 15.8, which is close to the average value of $n_{\mathrm{dr}}$ for all individual drainage curves, namely 16.3. The fact that normalizing the capillary pressure–saturation curves for drainage and imbibition yields one curve for drainage and one curve for imbibition implies that the variation in *n* can be neglected, and thus, *n* can be considered as a constant for the range of porosity values [0.10–0.43] and absorption values [5–40 g/g] that we considered in this study.

## Summary and Conclusions

In this research, a pore-scale model was developed to construct capillary pressure–saturation curves for swelling granular materials. The material of interest were particles of Absorbent Gelling Material (AGM). This model is based on combining the DEM and the pore-unit assembly method. It is capable of producing capillary pressure–saturation curves for different particle packings, based on simple physical parameters: particle size distribution, porosity, and, in case of swelling granular materials, the amount of absorbed water. In order to test the model, the capillary pressure–saturation curves were constructed for Hostun sand. They were found to be in relatively good agreement with experimental data from the literature. Then, a large number of capillary pressure-saturation curves for primary imbibition and drainage were constructed for different porosities and different amounts of absorbed water. The simulated data were used to develop a relationship for the Van Genuchten parameter $\alpha$ and *n* dependent on the amount of absorbed water and porosity. When we normalized the capillary pressure by the entry pressure $(1/\alpha )$ and plotted it against the effective saturation, we found that all drainage curves collapsed into one single drainage curve and all imbibition curves collapsed into one single imbibition curve, proving that both normalized curves are independent of porosity and absorption ratio. This implies that we can capture the effects of porosity and absorption ratio in the Van Genuchten parameter $\alpha$, maximum water saturation (during imbibition), and residual water saturation (during drainage) while keeping *n* constant.
